# Novel Cytotoxic Chemotherapies in Small Cell Lung Carcinoma

**DOI:** 10.3390/cancers13051152

**Published:** 2021-03-08

**Authors:** Diego Cortinovis, Paolo Bidoli, Stefania Canova, Francesca Colonese, Maria Gemelli, Maria Luisa Lavitrano, Giuseppe Luigi Banna, Stephen V. Liu, Alessandro Morabito

**Affiliations:** 1 Department Medical Oncology—ASST-Monza Ospedale San Gerardo, via Pergolesi 33, 20090 Monza, Italy; paolo.bidoli@unimib.it (P.B.); s.canova@asst-monza.it (S.C.); f.colonese@asst-monza.it (F.C.); maria.gemelli@asst-monza.it (M.G.); 2 School of Medicine and Surgery, University of Milano-Bicocca, 20900 Monza, Italy; marialuisa.lavitrano@unimib.it; 3 Department of Oncology, Portsmouth Hospitals University NHS Trust, Cosham, Portsmouth PO6 3LY, UK; giuseppe.banna@nhs.net; 4 Lombardi Comprehensive Cancer Center, Georgetown University, 3800 Reservoir Road NW, Washington, DC 20007, USA; stephen.v.liu@gunet.georgetown.edu; 5 SC Oncologia Medica Toraco-Polmonare, IRCCS Istituto Nazionale dei Tumori, Fondazione Pascale, 80100 Napoli, Italy; a.morabito@istitutotumori.na.it

**Keywords:** small cell lung cancer, chemotherapy, immunotherapy, lurbinectedin

## Abstract

**Simple Summary:**

Small cell lung cancer is a subtype of lung cancer and one of the deadliest thoracic tumours. Historically, chemotherapy consisting of either platinum plus etoposide or anthracycline-based regimens have been associated with a high response rate and rapid development of acquired resistance, contributing to the poor overall prognosis. Only a fraction of patients with local or early disease can be cured, whilst the treatment is palliative in those with extensive disease. In recent decades, few novel drugs have been developed, which are herein described.

**Abstract:**

Small cell lung cancer (SCLC) is one of the deadliest thoracic neoplasms, in part due to its fast doubling time and early metastatic spread. Historically, cytotoxic chemotherapy consisting of platinum–etoposide or anthracycline-based regimens has demonstrated a high response rate, but early chemoresistance leads to a poor prognosis in advanced SCLC. Only a fraction of patients with limited-disease can be cured by chemo-radiotherapy. Given the disappointing survival rates in advanced SCLC, new cytotoxic agents are eagerly awaited. Unfortunately, few novel chemotherapy drugs have been developed in the latest decades. This review describes the results and potential application in the clinical practice of novel chemotherapy agents for SCLC.

## 1. Introduction

Small cell lung cancer (SCLC) is an aggressive neuroendocrine tumour, accounting for 13–15% of new lung cancer diagnoses with a lower prevalence among lung cancer due to short survival. It is characterised by rapid cellular proliferation, deregulation of cell cycle control and apoptosis and high chemosensitivity followed by quick emergence of resistance to many therapies [1]. Cytotoxic chemotherapy currently represents the standard treatment to reduce tumour growth and limit metastatic spread [2], though its benefit is consistently transient. Chemotherapy given with radiotherapy can cure the limited-disease (LD) SCLC, but this is still only achieved by a fraction of patients, with a 5-year survival rate of 10–20% [3]. For patients with extensive-disease (ED) SCLC, chemotherapy has only a palliative intent, with anecdotal long-term survivors following this treatment but survival for most patients limited to 8–10 months.

Since 1985, the combination of platinum plus etoposide has been the standard treatment for both the LD- and ED-SCLC [4,5]. The superiority of regimens containing platinum-derivatives, as compared to non-platinum regimens, has been confirmed in several meta-analyses [6,7,8]. Carboplatin and cisplatin have equivalent activity and efficacy in SCLC; however, carboplatin has a more favourable toxicity profile than cisplatin, with the exception of more myelosuppression activity [9].

Beyond platinum and etoposide, other combinations have been tested with response rates ranging from 50 to 60%, median progression-free survival (PFS) and overall survival (OS) of 4–5 months and 8–12 months, respectively. Specifically, the combination of cisplatin and irinotecan showed a superior efficacy than cisplatin and etoposide in the Asiatic population [10], whereas combining cisplatin with topotecan or an anthracycline did not result as more effective as to etoposide [11,12].

Other attempts to exploit the chemosensitivity included dose intensification and the use of non-cross-resistant drugs with different mechanisms of action, which did not prove to be superior to the platinum and etoposide combination; the high-dose chemotherapy followed by bone marrow transplant was abandoned due to the lack of improved long-term survival despite the high incidence of serious adverse events [13,14,15,16,17,18,19]. Non-cross resistant chemotherapy agents are used to treat disease relapse in the second-line setting, with variable results depending on the treatment-free interval (i.e., > or ≤90 days from the end of the first-line therapy) [20].

For many decades, topotecan was the only FDA (Food and Drug Administration) and EMA (European Medicine Agency) approved drug for patients progressing to the first-line therapy based on an equivalent efficacy and better tolerability compared to the triplet of cyclophosphamide, doxorubicin and vincristine (i.e., the CAV regimen) [21]. In a randomised clinical trial, single-agent topotecan showed a median PFS of 2 months less than carboplatin and etoposide (2.7 vs. 4.7 months, respectively) in patients with a treatment-free interval >90 days; the mOS, however, was not different between the two arms [22]. In June 2020, lurbinectedin received accelerated FDA approval.

Since the end of the 1990s, the clinical recommendations on systemic chemotherapy had not changed. No drugs with novel mechanisms of action were available, other than alkylating agents or DNA intercalating drugs. Numerous clinical trials with conventional drugs failed to demonstrate any outcome improvement, and the efficacy of platinum plus etoposide was not surpassed, despite its modest results. This generated a basic nihilism that did not facilitate progress.

In recent years, some clinical trials paved the way for the development of new drugs with different mechanisms of action and new combinations for the SCLC.

More recently, the advent of immunotherapy has brought about new lifeblood in research applied to SCLC. In particular, in the first line, the increase in overall survival highlighted with the addition of anti-programmed-cell-death-1 (antiPD-1) and anti-PD-ligand-1 (antiPD-L1) led to a change in the standard of care. For this reason, the association of platinum and etoposide for 4–6 cycles can no longer be considered the gold standard. In fact, even in these more recent randomised studies, the standard chemotherapy arm resulted in OS between 9.7 and 10.5 months, comparable to what combination chemotherapy treatment has shown for about 40 years.

In further lines, on the contrary, chemotherapy treatment remains a standard of care, although the results in terms of clinical outcomes are unsatisfactory.

The purpose of this review is to discuss new chemotherapeutics and how old chemotherapeutics may have a new life through innovative approaches.

## 2. Older Chemotherapeutics as a Companion to Immune-Checkpoint Inhibitors (ICIs): New Insights to Exploit Synergy

In the last decade, immunotherapy significantly improved the clinical outcomes of patients with thoracic malignancies. The use of immunotherapy in ED-SCLC holds its rationale on the high mutational rate and chemotherapy tumour cell killing effect, potentially associated with the expression and release, respectively, of a high number of neoantigens. This could elicit and enhance the activity of ICIs, translating into a clinical benefit [23].

Three phase III studies investigated the combination of chemotherapy and ICIs in the ED-SCLC: the CASPIAN study, the IMpower 133 and the KEYNOTE-604 [24,25,26] (Table 1).

The CASPIAN study randomised patients with ED-SCLC in a 1:1:1 ratio to receive durvalumab plus platinum–etoposide, durvalumab plus tremelimumab plus platinum–etoposide, or platinum–etoposide alone. The updated analysis showed an improvement in the mOS in the durvalumab plus platinum–etoposide arm as compared to chemotherapy alone (12.9–95% confidence interval [CI] 11.3–14.7–versus 10.5 months–95% CI 9.3–11.2–respectively), with 22.2 versus 14.4% of patients, respectively, alive at 24 months. The addition of tremelimumab to durvalumab and platinum and etoposide did not improve survival compared to chemotherapy alone [24]. The IMpower133 study randomised in a 1:1 ratio of patients with ED-SCLC to atezolizumab plus carboplatin–etoposide or placebo plus carboplatin-etoposide. The addition of atezolizumab improved both the mOS (12.3 vs. 10.3 months, respectively, hazard ratio [HR] for death 0.70, 95% CI 0.54–0.91, *p* = 0.007) and median PFS (5.2 vs. 4.3 months, HR for progression 0.77, 95% CI 0.62–0.96, *p* = 0.02) [25]. The KEYNOTE-604 study randomised in a 1:1 ratio the addition of pembrolizumab to platinum–etoposide vs. placebo plus platinum–etoposide in patients with ED-SCLC. The addition of pembrolizumab statistically improved only the median PFS (4.5 vs. 4.3 months, HR 0.75, 95% CI 0.61–0.91, *p* = 0.023), whereas the median OS was not significantly prolonged according to the prespecified criteria for statistical significance [26].

These studies have similar design and survival primary endpoints, with key differences regarding the use of a programmed-cell-death-1 (PD-1) inhibitor (instead of a PD-ligand-1 (PD-L1)) in the KEYNOTE-604 study, the choice of the carboplatin as the only platinum compound in the IMpower 133 study, and the open-label design in CASPIAN study. Overall, a durable survival benefit emerged from the combination of ICIs with chemotherapy. Atezolizumab received FDA approval in March 2019 and EMA approval in July 2019, whereas durvalumab was approved by FDA in March 2020, both as first-line therapy for ED-SCLC [27]. Regarding safety, the immune-related adverse events (irAEs) from the chemo-immunotherapy in ES-SCLC had a mild impact on the overall toxicity, without any significant difference in grade 3–4 AEs as compared to the chemotherapy [28].

Despite the favourable outcomes of chemo-immunotherapy observed in clinical trials, their translation into the clinical practice has some limitations. For instance, patients with severe comorbidities, frailties and/or an Eastern Cooperative Oncology Group (ECOG) Performance Status (PS) ≥2 were excluded from clinical trials, although they represent up to 30 to 40% of patients with ED-SCLC [29]. Furthermore, only those patients with stable brain metastases were enrolled in clinical trials. Consequently, the proportion of patients with brain disease treated in clinical trials with chemo-immunotherapy (10.4% overall) was smaller than that observed in clinical practice (which is up to 24%) [28]. Finally, no predictive biomarker for chemo-immunotherapy is still available in ED-SCLC. As demonstrated in the CASPIAN and IMpower 133 studies, neither PD-L1 expression nor tumour mutational burden (TMB) using various thresholds was predictive for efficacy from the addition of immunotherapy to the chemotherapy [30,31].

It is also noteworthy the lack of an obvious effect from ICIs on the median duration of response (DOR) observed across the different above-mentioned studies and in contrast with evidence in other cancers. One possible explanation for this effect is that a high overall response rate (ORR) is already achieved with the chemotherapy in SCLC, as for the high chemosensitivity of this cancer. In addition, the short follow-up duration of the studies might have hidden the gain in the median DOR from the addition of the immunotherapy. Indeed, when a landmark endpoint such as the 12-month ORR was considered, the benefit from ICIs in terms of response duration was detectable [32].

These considerations raise another important point regarding the optimal chemotherapy backbone for immunotherapy. The chemotherapy and chemo-immunotherapy arms PFS and OS curves separated after 4–7 months in clinical trials, which might suggest the lack of a synergistic effect between platinum–etoposide and ICIs. In experimental models, this lack of synergy was explained by a mechanism involving calreticulin (CALR) in immunogenic cell death. CALR is a protein normally located in the endoplasmic reticulum; it translocates to the cell membrane in response to the endoplasmic reticulum (ER) stress and provides an “eat-me” signal to antigen-presenting cells. The anthracyclines, but not etoposide, are efficient immunogenic cell death inducers and strongly immunogenic in preclinical mouse models [23]. In the phase I CheckMate 012 study on patients with non-small-cell-lung cancer (NSCLC), nivolumab was evaluated in three different chemotherapy regimens, including gemcitabine–cisplatin (for the squamous histology) or pemetrexed–cisplatin (for the nonsquamous tumours) or paclitaxel–carboplatin (for all the histologies). Although it was for a limited number of patients, the combination of nivolumab 5 mg/kg with paclitaxel–carboplatin yielded a similar overall response rate and higher 24-month OS than pemetrexed–cisplatin [33]. Further studies are needed to confirm whether a different chemotherapy backbone and platinum-containing regimens than platinum–etoposide may produce more favourable outcomes in ED-SCLC by the addition of the immunotherapy. A combination of lurbinectedin and atezolizumab is currently ongoing in a phase I/II study, with atezolizumab at a fixed dose of 1200 mg followed by lurbinectedin at a starting dose of 3.2 mg/m^2^ as a 1-hour infusion on day 1, every three weeks, in patients with SCLC progressing on first-line platinum-based chemotherapy (NCT04253145).

Furthermore, there is a renewed interest in developing new platinum compounds by exploiting the recent knowledge on the sensitivity and resistance mechanisms of cancer cells, their epigenetic modifications, which translate into a platinum drug-tolerant cancer phenotype, and the immunomodulatory effects of platinum compounds to limit immune cell exhaustion [34].

## 3. Oral Versus Intravenous (i.v.) Formulations of Chemotherapy

The severe acute respiratory syndrome coronavirus 2 (SARS-CoV-2) pandemic has been challenging the oncology services and how we currently administer systemic treatments to patients with cancers, particularly in the palliative setting [35]. Patients with lung cancer could represent a vulnerable population to this infection [36,37,38] with high related mortality in the range of 25–39% [36,37,38,39,40,41,42].

Oral alternatives to i.v. anticancer therapies have gained attention, given efforts to reduce visits to the hospitals and the associated risk of infective transmission [42,43]. There is also a benefit in terms of patient convenience and preference with PO (per os) anticancer drugs, provided that their efficacy is equivalent to their i.v. counterparts [44].

Oral formulations of either topotecan and etoposide, two of the most active and used drugs in SCLC, are available and have the following sufficient data to support their use.

### 3.1. Oral Topotecan

Oral topotecan (2.3 mg/m^2^/d, from day 1 to day 5, every 21 days) has demonstrated an absolute benefit in OS (of 12 weeks), slower quality of life deterioration, and greater symptom control, as compared to best supportive care (BSC) by a phase III randomised clinical trial in 141 patients with relapsed SCLC, regardless of their treatment-free interval (< or ≥60 days) [45].

As a second-line treatment, a phase III randomised trial comparing oral topotecan (2.3 mg/m^2^/d from day 1 to day 5) with i.v. topotecan (1.5 mg/m^2^/d from day 1 to day 5, every 21 days) demonstrated similar activity and tolerability between the two formulations in 309 patients with SCLC sensitive to initial chemotherapy (i.e., with a treatment-free interval of > or = 90 days). The absolute difference in response rates between oral and i.v. topotecan was −3.6% (18.3 vs. 21.9% for oral and i.v., respectively), whilst no difference in OS was observed. The toxicity profile was different with more thrombocytopenia and diarrhea for oral topotecan, but less neutropenia and anemia, as compared to i.v. topotecan [46]. Another phase III randomised trial compared oral topotecan (2.3 mg/m^2^ from day 1 to day 5) every 21 days for six cycles to carboplatin (area under the curve 5 mg/mL per min day 1) plus etoposide (100 mg/m^2^ from day 1 to day 3), as a second-line treatment for 164 patients with sensitive relapsed (at least 90 days after completion of first-line treatment) SCLC [22]. The median PFS was significantly longer in the combination chemotherapy group than in the oral topotecan group with an absolute benefit of 2.0 months (HR 0.57, 90% CI 0.41–0.73; *p* = 0.0041), but no OS difference was observed (HR 1.03, 95% CI 0.87–1.19; *p* = 0.94). The toxicity was comparable between the two groups, although two treatment-related deaths occurred in the oral topotecan group (both were febrile neutropenia with sepsis) as compared to none in the combination group.

As first-line treatment, a phase III study randomised 784 patients with untreated ED-SCLC to either oral topotecan (1.7 mg/m^2^/d from day 1 to day 5) with i.v. cisplatin (60 mg/m^2^ on day 5) (TC) or i.v. etoposide (100 mg/m^2^/d from day 1 to day 3) with i.v. cisplatin (80 mg/m^2^ on day 1) (PE) every 21 days. No difference in OS was observed between the two groups with the absolute difference of −0.03 (95% CI, −6.53 to 6.47), meeting the predefined criteria for non-inferiority of TC relative to PE. The regimens were similarly tolerable, with more frequent grade 3/4 neutropenia with PE (84% vs. 59%), grade 3/4 anemia and thrombocytopenia with TC (38 vs. 21% and 38 vs. 23%, respectively) [11].

### 3.2. Oral Etoposide

As an alternative to the i.v. formulation, the use of oral etoposide is supported by randomised data and a registry real-world population-based study of 2066 chemotherapy naïve patients with LD- (*n* = 762) and ED-SCLC (*n* = 1264) [47]. These studies showed, both in the LD- and in the ED-SCLC, no significant difference in OS between i.v. versus oral etoposide, although the oral group did require more dose reductions as compared to the i.v. group [47]. The oral etoposide was given on days 2 and 3 at the doubled dose of 200 mg/m^2^ than 100 mg/m^2^ of the i.v.

### 3.3. Conclusions

Based on these data, oral topotecan and etoposide could be considered as convenient alternatives to other i.v. therapies either in first-line, as a substitute for i.v. etoposide in platinum-combinations, and, for the oral topotecan, in subsequent treatment lines, as compared to other i.v. options, particularly for the platinum-resistant disease. Furthermore, the reduced incidence and severity of neutropenia observed with oral topotecan in two of the three above-mentioned phase III studies, either when compared to the i.v. etoposide and to its i.v. formulation, may represent a further advantage during the SARS-CoV-2 pandemic and speculatively for novel chemo-immunotherapy combinations.

## 4. Lurbinectedin

Lurbinectedin is an oncogenic transcription inhibitor. The drug is an analogue of trabectedin which covalently binds guanine residues in the minor groove of DNA, creating adducts that are able to induce double-strand breaks. As a consequence, a cascade of events occurs affecting the activity of DNA binding proteins, involving transcription factors and DNA repair mechanisms, leading to double-strand breaks and finally to cell death [48]. According to its mechanism of action, lurbinectedin showed antiproliferative and cytotoxic functions in several tumour cell lines and increased activity in cell lines bearing defects in the DNA mismatch repair. Moreover, lurbinectedin causes ICD (immunogenic cell death) and elicits anticancer immunity [49]. A single preclinical study demonstrated a direct effect of lurbinectedin on the tumour microenvironment, as it decreases the tumour-associated macrophages and circulating monocytes, and the angiogenic factor VEGF (vascular endothelial growth factor), with consequent reduced blood vessel density [50]. Lurbinectedin received accelerated approval by the FDA in 2020 for adult patients with metastatic SCLC progressing on or after platinum-based chemotherapy after demonstrating favourable ORR and DOR in an open-label, single-arm phase II trial [51] (Table 2).

## 5. Clinical Development

### 5.1. Phase I Trials

A phase I study indicated the dose of 4 mg/m^2^ or a flat dose of 7 mg i.v. every 21 days as safe dosing for lurbinectedin [57]. Based on preclinical data of synergy, another phase I study investigated the combination of lurbinectedin at 4 mg flat dose with doxorubicin 50 mg/m^2^ [53]. The study enrolled 27 relapsed SCLC patients. When administered as second-line, the combination showed relevant activity as 91.7% of patients with a platinum-sensitive disease (defined as with platinum-free interval ≥ 90 days) and 33.3% of those with a platinum-resistant disease (with platinum-free interval < 90 days) achieved a disease response, with a PFS of 5.8 and 3.5 months, respectively. As a third-line treatment, all patients had a platinum-resistant disease, and the median PFS was 1.2 months. The major toxicities were hematologic, with high rates of grade 3–4 neutropenia (95%), leukopenia (79%), anemia (47%) and thrombocytopenia (26%). Aiming at improving the safety of this combination, an expansion cohort with a reduced dose of both drugs (lurbinectedin 2 mg/m^2^ and doxorubicin 40 mg/m^2^) was implemented, with an observed ORR of 37% and 53% in patients with a resistant and sensitive disease, respectively. The median PFS was 3.4 months in all patients, 1.5 and 5.7 months in those with a resistant and sensitive disease, respectively. The median OS was 7.9 months in all patients, 4.9 and 11.5 months in those with a resistant and sensitive disease, respectively [58].

Another combination with lurbinectedin and irinotecan has been explored in a phase Ib/II trial for advanced solid tumours (NCT02611024). Patients with SCLC included in this study were 13. The study investigates an escalating dose of lurbinectedin starting from 1 mg/m^2^ on day one with a fixed dose of irinotecan 75 mg/m^2^ on days 1 and 8, every 21 days. The recommended dose of lurbinectedin was 2.0 mg/m^2^, and the maximum tolerated dose was 2.4 mg/m^2^ in combination with irinotecan 75 mg/m^2^ and prophylactic granulocyte-colony stimulating factor (G-CSF). In the SCLC cohort, the ORR was 61.5% [54,56].

### 5.2. Phase II Trial

Recently, the results of a phase II, open-label, basket trial exploring the safety and efficacy of lurbinectedin as a single-agent in several tumour types were published, including 105 patients with progressive SCLC [52]. The eligibility criteria required previous platinum-based chemotherapy and the absence of central nervous system (CNS) involvement. Forty-five and 60 patients had chemotherapy-resistant and -sensitive disease, respectively, with a chemotherapy-free interval of ≥90 days. The median follow-up was 17.1 months. The ORR was 35.2% (95% CI, 26.2–45.2), the median DOR and mOS were 5.3 and 10.8 months, respectively, in all SCLC patients. Patients with platinum-sensitive disease showed better outcomes as compared to platinum-resistant disease, with a median DOR of 6.2 months (95% CI, 3.5–7.3) and 4.7 months (95% CI, 2.6–5.6), respectively. The most frequent grade 3–4 AEs were hematological. Furthermore, a preplanned subset analysis of patients with chemotherapy-free interval ≥ 180 days was conducted on 20 patients. The ORR was 60% (95% CI, 36.1–86.9) in these patients, with a median DOR of 5.5. months (95% CI, 2.9–11.2) and a disease control rate (DCR) of 95% (95% CI, 75.1–99.9). The median PFS was 4.6 months (95% CI, 2.6–7.3), and the median OS 16.2 months (95% CI, 9.6-not reached). The most common grade 3–4 adverse events were hematological and increased liver function tests [59].

### 5.3. Phase III Trial

The phase III ATLANTIS study (NCT02566993) compared the combination of lurbinectedin and doxorubicin to topotecan or CAV at the physician’s choice. Sixty-hundred-thirteen patients with SCLC progressing to one prior platinum-based chemotherapy were randomised 1:1. Lurbinectedin was given at 2 mg/m^2^, lower than that of 3.2 mg/m^2^ approved by the FDA. Patients were stratified by their chemotherapy-free interval, ECOG PS of 0 or 1–2, CNS involvement, prior use of ICIs (anti-PD-1/PD-L1 agents), and investigator’s preference of topotecan or CAV. The recent press release from Jazz Pharmaceuticals and PharmaMar announced that the experimental treatment with lurbinectedin and doxorubicin missed its prespecified OS endpoint [55]. The secondary outcome endpoints and subgroup analyses, however, favoured the investigational combination in the intent-to-treat patient population, including the analysis of OS differences between the subgroup of patients treated with lurbinectedin and doxorubicin versus CAV; the OS and PFS in patients with and without CNS metastases; the ORR and DOR as assessed by an independent review committee.

Patients with relapsed SCLC still represent an unmet medical need as they have limited treatment options, lack of druggable targets, and poor prognosis. Despite the innovative mechanism of action and promising phase I and II trial results, lurbinectedin in combination with doxorubicin did not meet its prespecified OS endpoint in the phase III trial. As above-mentioned, however, there is still rationale and room for exploring combinations of lurbinectedin with ICIs +/− other chemotherapy agents, based on its immunomodulatory effects. (Table 2).

## 6. Novel Formulations of Traditional Chemotherapy Agents and “Promising” Drug Derivatives

Few cytotoxic agents with modest benefit and poor tolerability are currently available for SCLC, especially in later treatment lines. Although potentially associated with prolonged survival, their toxicity profile often represents a barrier to their administration at the full recommended dose. In addition to new agents with a different mechanism of action, the development of newer formulations of traditional chemotherapy drugs might represent a promising strategy to improve either activity and safety (Table 3).

### 6.1. Nanoparticle Albumin Bound-Paclitaxel (Nab-Paclitaxel)

Paclitaxel, alone or in combination with carboplatin, has shown activity in the refractory relapsed SCLC; however, its use is limited by potentially severe infusion reactions and peripheral neurotoxicity [60,61,62]. A solvent-free formulation of paclitaxel, the nanoparticle albumin-bound (nab)-paclitaxel, has been developed to improve the tolerability of paclitaxel, confirming efficacy and a safer profile than paclitaxel in pancreatic, breast cancer and NSCLC [63]. The activity of single-agent weekly nab-paclitaxel was tested in phase II, single-arm, NABSTER trial on 68 patients with relapsed SCLC, divided into two cohorts based on platinum-sensitivity (with a cut-off of 60 days). The primary endpoint was investigator-assessed ORR; PFS and OS were secondary endpoints. The RR was 8% and 14% in the refractory and sensitive cohort, respectively. The median PFS was similar in both the cohorts (1.8 and 1.9 months), whilst the median OS was longer in the sensitive than in the refractory cohort (6.6 vs. 3.6 months, respectively). The treatment was well tolerated with grade 3–4 neutropenia in 10% of patients and anemia in 4%. The study did not meet the primary endpoint on ORR, and the authors concluded that further investigations and a head-to-head comparison with topotecan were not justified [64].

### 6.2. Liposomal Irinotecan (Nal-IRI)

Liposomal formulations of chemotherapy agents, such as irinotecan and topotecan, have been developed to improve their efficacy through a slow and controlled release of the drug expected to prolong the exposure of tumour cells to these agents [65,66]. Irinotecan is active in the first-line treatment of SCLC in combination with cisplatin and is commonly used in Japan. In the second-line setting, single-agent irinotecan showed similar outcomes to those from topotecan and is included in several guidelines as a possible treatment option [67,68,69].

The liposomal encapsulation of irinotecan has been designed to prolong its circulation levels, exploiting the tumour vascular permeability and accumulation in the tumour tissue, where the macrophages can activate the drug [65]. Compared to irinotecan, its liposomal formulation demonstrated a longer persistence of plasma levels of SN-38, the active metabolite (50 vs. 8 h in mice), resulting in sustained topoisomerase-1 inhibition, increased DNA damage and cell death [70].

In metastatic pancreatic cancer, liposomal irinotecan in combination with fluorouracil and folinic acid showed significant improvement in OS in phase III Napoli-1 trial and was approved by the FDA and EMA as second-line treatment after failure of gemcitabine-based chemotherapy [71].

SCLC is highly vascularised and enriched by tumour-associated-macrophages (TAM). These two characteristics suggest a possible high penetration of liposomal irinotecan (nal-IRI) in the tumour tissue and activation by local phagocytic through high CES (carboxylesterase) levels, the enzyme that converts irinotecan in its active metabolite SN-38, resulting in effective cytotoxic activity [72,73,74]. In preclinical models, nal-IRI showed superior antitumour activity than topotecan and irinotecan, either in cell-line derived models and patients-derived xenograft models built after progression to carboplatin and etoposide [75]. These preclinical data supported the clinical development of nal-IRI in patients with SCLC.

The RESILIENT study is an ongoing two-part phase II/III trial assessing the potential use of nal-IRI in patients with SCLC who progressed on or after platinum-based regimens. Part one of the trial involved dose-finding and dose-escalation analyses, whilst in the second part, patients were randomised to liposomal irinotecan or topotecan to compare efficacy in terms of PFS and OS. In the dose-finding phase, patients were divided into two cohorts to receive liposomal irinotecan every 2 weeks at 70 mg/m^2^ or 85 mg/m^2^. The cohort treated with 85 mg/m^2^ dose was closed early for dose-limiting toxicities. In the second cohort, 12 patients received the 70 mg/m^2^ dose, which was deemed by the investigators as tolerable. Among these patients, preliminary exploratory efficacy endpoints were promising, with a RR of 33.3%, the median time to response of 6 weeks and an overall DCR of 58.3% [76]. This led to a dose-expansion phase with additional 13 patients, whose safety data were presented at the 2020 World Conference on Lung Cancer (WCLC). Among the 25 patients who received the 70 mg/m^2^ dose, the grade ≥3 treatment-emergent AEs occurred in 40% (*n* = 10) of patients, mostly represented by diarrhea (*n* = 5), neutropenia (*n* = 4), anemia (*n* = 2) and thrombocytopenia (*n* = 2) [77]. The trial is still recruiting for the second part (www.ClinicalTrials.gov Identifier: NCT03088813; accessed on 20 January 2021).

### 6.3. Belotecan

Belotecan is a new camptothecin analogue and topoisomerase I inhibitor. Preclinical data on mice models suggested superior antitumour efficacy and wider therapeutic margins than topotecan [78]. In phase II clinical trials, belotecan monotherapy showed encouraging activity and good tolerability in patients with relapsed SCLC [79,80]. More recently, second-line belotecan has been compared to topotecan in a phase IIb trial on 164 SCLC patients progressing to platinum-based chemotherapy. Patients were randomised in a 1:1 ratio to receive five consecutive daily intravenous infusions of topotecan (1.5 mg/m^2^) or belotecan (0.5 mg/m^2^) every 3 weeks for six cycles. The study was powered to assess the non-inferiority of belotecan to topotecan in ORR. Belotecan significantly improved ORR (33 vs. 21%, respectively, *p* = 0.09) and DCR (85 vs. 70%, *p* = 0.030) as compared to topotecan. Furthermore, the median OS was significantly longer with belotecan than topotecan (13.2 vs. 8.2 months, HR = 0.69, 95% CI 0.48–0.99), with a favourable safety profile. On the basis of these promising results, belotecan might be another treatment option for second-line treatment in SCLC, pending phase III trials to confirm its efficacy in this setting [81].

### 6.4. Amrubicin

Amrubicin is a third-generation synthetic anthracycline with potent inhibiting activity on the topoisomerase II. Among drugs belonging to the same class, amrubicin has fewer chronic cardiological effects (e.g., cardiomyopathy) and no cumulative-dose heart damage in animal models [82]. Amrubicin is approved in Japan as a single-agent for SCLC after the failure of platinum-based chemotherapy, whilst it is under evaluation in other countries. In this setting, it has been investigated by several studies on Asian patients, showing ORR ranging from 36 to 52% and median OS of 7–12 months [83,84,85,86,87]. Two phase II studies demonstrated clinical efficacy in the Caucasian population also, with higher ORR as compared to topotecan (44 vs. 15%, respectively) [88,89]. A phase III study compared amrubicin with topotecan in patients with SCLC progressing to platinum–etoposide chemotherapy. A total of 637 patients were randomly assigned in a 2:1 ratio to receive amrubicin 40 mg/m^2^ on days 1–3 every three weeks or topotecan 1.5 mg/m^2^ on days 1–5 of 21 days cycles. Amrubicin did not improve the primary endpoint of OS as compared to topotecan (7.5 vs. 7.8 months, HR 0.88, *p* = 0.170), despite an improvement in the median PFS (4.1 vs. 3.5 months, HR, 0.80, *p* = 0.018) and ORR (31.1 vs. 16.9%, odds ratio [OR] 2.22, *p* < 0.001). A slight survival benefit of two weeks was observed in the subgroup of patients with refractory disease. The safety profile favoured amrubicin as far as hematologic events are concerned; however, higher rates of infections (16 vs. 10%, respectively) and febrile neutropenia (10 vs. 3%, respectively) were linked to amrubicin [90].

In the first-line setting of ED-SCLC, the combination of amrubicin plus cisplatin has been compared to cisplatin–etoposide in phase III non-inferiority study in Chinese patients. The amrubicin–cisplatin regimen was non-inferior to standard platinum–etoposide chemotherapy on OS (median OS of 11.8 vs. 10.3 months, *p* = 0.08), with a slight non-significant improvement of 1.5 months [91].

The potential interest of this drug derives from its possible synergy with other agents. Although the results of the lurbinectedin/doxorubicin combination showed by the ATLANTIS trial press release were disappointing [55], amrubicin still remains a plausible alternative to the doxorubicin as a potential companion drug for lurbinectedin in future clinical trials. Furthermore, based on its immunomodulatory effect, the association of amrubicin and pembrolizumab is under evaluation in a phase II trial in patients with refractory SCLC [92]. Therefore, despite the modest activity showed in non-Asian patients as single-agent, amrubicin may still have a role as a new companion for combination strategies in clinical trials.

### 6.5. Temozolomide

Temozolomide is an oral alkylating agent that induces cytotoxic damage and apoptosis through single-strand DNA breaks. The rationale for its application in SCLC is strong, as temozolomide has a good penetration through the blood–brain barrier, which can be useful for brain metastasis, and SCLC has aberrantly methylated O6-methylguanine-DNA methyltransferase MGMT, the enzyme involved in the repair mechanism of the DNA damage induced by temozolomide [93].

Two phase II single-arm studies investigated the role of the single agent temozolomide in SCLC patients who progressed to one or two lines of chemotherapy, stratified on the basis of platinum sensitiveness. In the first by Pietanza et al., 64 patients were enrolled and received temozolomide 75 mg/m^2^/die for 21 days in a 28-day cycle. The primary endpoint was ORR in the platinum-sensitive and refractory cohort. The ORR was 22% (95% CI, 9–40%) and 19% (95% CI, 7–36%), respectively. One complete remission was observed in the platinum-sensitive cohort. The main limiting toxicities were grade 3 thrombocytopenia and neutropenia, which were observed in nine patients (14%) [94].

In the second study by Zauderer MG et al., a different schedule was used to improve tolerability on hematologic toxicities. Temozolomide was given 200 mg/m^2^/die for 5 days in a 28-day cycle. Among 25 SCLC patients, five patients have grade 3–4 events (mostly anemia, thrombocytopenia and neutropenia). The ORR was 12% (95% CI 3–31%), with two responses also observed in refractory patients, median PFS was 1.8 months (95% CI 0.9–3.5 months) and median OS 5.8 months (95% CI: 3.3–9.8 months) [95].

Data on brain metastasis response were conflicting among the two studies. In the first study, at the standard dose, 38% had a CR (complete response) or PR (partial response) (95% CI, 14–68%) [94], whilst no response was seen in the eight patients with target brain lesions of the second study (four had stable disease and four progression) [95].

MGMT promoter methylation is a well-known predictive factor of response to temozolomide in glioma [96]. For this reason, it was evaluated in both the aforementioned studies. In the first one, ORR improved in patients with methylated MGMT promoter with respect to patients without methylation (38 vs. 7%; *p* = 0.08) [94]. In the second one, 50% of patients had methylation in MGMT promoter, but the number of patients was too small to derive statistical conclusions on response [95].

However, due to the scarce data on efficacy and hematologic toxicity, temozolamide single agent is not routinely used in clinical practice.

More recently, the association of temozolamide with PARP (poly ADP-ribose polymerase)-inhibitors, veliparib and olaparib, has been investigated [97,98]. Alterations in the PARP-dependent base excision repair pathway are an established resistance mechanism to temozolamide, and preclinical models validated the rationale for the clinical development of combinations with temozolamide and PARP-inhibitors [99]. The combination of temozolamide plus veliparib or olaparib improved ORR compared to temozolamide alone in phase II trials [97,98]. However, data on larger cohorts and phase III trials are ongoing to assess the possible application in clinical practice.

## 7. Conclusions

After many years of inactivity, the treatment of SCLC has been observing a renewed interest thanks to the introduction of immunotherapy in the first-line setting.

Cytotoxic agents remain so far the backbone treatment for immunotherapy in the first-line and the only current options in later therapeutic lines, although limited benefit and relevant toxicity, particularly in the platinum-resistant population.

Re-interpreting and exploiting the mechanisms of action of old cytotoxic agents, such as cisplatin and anthracyclines, with a view to their immunomodulatory effects, can unveil new therapeutic scenarios with combination strategies based on ICIs and different chemotherapeutic agents or their new formulations.

Despite the limitations highlighted by all the chemotherapy molecules currently investigated, the discovery of transcription factors and their overexpression will allow the segmentation of SCLC into different molecular subgroups, which could benefit from combinations of chemotherapy with other small molecules (such as PARP inhibitors, Aurora kinase inhibitors).

The chemotherapy chapter for SCLC is not yet closed, and new studies are needed to better understand its role in these future therapeutic scenarios.

## Figures and Tables

**Table 1:** Phase III trial results of chemotherapy + immune checkpoint inhibitors. Abbreviations: mOS = median Overall Survival; mPFS = median Progression Free Survival; HR = Hazard Ratio.

First Line-Chemo-Immunotherapy Trials	IMpower 133		CASPIAN			KEYNOTE 604	
	Atezolizumab	Placebo	Durvalumab	Durvalumab Tremelimumab	Placebo	Pembrolizumab	Placebo
mPFS, mos	5.2	4.3	5.1	4.9	5.4	4.5	4.3
HR (95%)	0.77 (0.63–0.95)		0.78 (0.65–0.94)	0.84 (0.7–1.01)		0.75 (0.61–0.91)	
mOS, mos	12.3	10.3	12.9	10.4	10.5	10.8	9.7
HR (95%)	0.70 (0.54–0.91)		0.75 (0.62–0.91)	0.82 (0.68–1)		0.8 (0.64–0.98)	
12-mos OS, %	51.9	39	52.8	43.8	40	45.1	39.6
24-mos OS, %	22	16.8	22.2	23.4	14.4	22.5	11.2

**Table 2:** Lurbinectedin trials on small cell lung cancer (SCLC). Median progression-free survival (mPFS) and median overall survival (mOS) expressed in months, overall response rate (ORR) as a percentage, with 95% CI in graphs. Abbreviations: N/A = not available; mOS = median Overall Survival; mPFS = median Progression Free Survival; ORR = Overall Response Rate; ITT = Intent To Treat population.

Ref	Phase	*N*	Intervention	ITT			Platinum Sensitive			Platinum Refractory		
				mOS	mPFS	ORR	mOS	mPFS	ORR	mOS	mPFS	ORR
[52]	II	105	Lurbinectedin 3.2 mg/m2 1q21	9.3 (6.3–11.8)	3.5 (2.6–4.3)	35.2% (26.2–45.2)	11.9 (9.7–16.2)	4.6 (2.8–6.5)	45.0% (32.1–58.4)	5.0 (4.1–6.3)	2.6 (1.3–3.9)	22.2% (11.2–37.1)
[53]	I	27	Doxorubicin 50 mg/m^2^ Lurbinectedin 4.0 mg (dose escalation from 3.5 mg) 1q21	7.9 (5.0–12.0)	4.1 (1.4–5.8)	57.7% (36.9–76.6)	11.5 (13.5–8.5)	5.8 (3.6–10.9)	91.7% (61.5–9.8)	4.9 (7.3–2.8)	3.5 (1.1–8.0)	33.3% (7.5–70.1)
[54]	Ib/II	13	Irinotecan 75 mg/m^2^ 1,8q21 lurbinectedin 2.0 mg day 1q21 (dose escalation from 1.0 mg)	N/A	5.4	61.5%	N/A	N/A	N/A	N/A	N/A	N/A
[55]	III	613	Lurbinectedin 2.0 + Doxorubicin 40.0 mg1q21 versus cyclophosphamide + doxorubicin + vincristine (CAV) versus topotecan	N/A	N/A	50%	N/A	N/A	N/A	N/A	N/A	N/A
[56]	Ib/II	7	Paclitaxel 80 mg/mq 1,8q21 + lurbinectedin 2.2 mg day 1q21 (dose escalation from 1.0 mg)	N/A	4.8	71%	N/A	N/A	N/A	N/A	N/A	N/A

**Table 3:** Novel formulations of traditional chemotherapy and drug derivatives clinical trials results. mPFS and mOS expressed in months, ORR as percentage, with 95% CI in graphs. Abbreviations: ITT = Intent To Treat population; N/A = not available; mOS = median Overall Survival; mPFS = median Progression Free Survival; ORR = Overall Response Rate.

Drug	Phase	Intervention	ITT			Toxicities (G3–4 AEs)
			mOS	mPFS	ORR	
**Nab-paclitaxel**	II (NABSTER)	Nab-paclitaxel 100 mg/mq die 1–8-15q28	3.65 refractory 6.64 sensitive	1.84 refractory 1.88 sensitive	11.8%	Fatigue (54%) Anaemia (38%) Neutropenia (29%) Leukopenia (26%) Diarrhea (21%)
**Liposomal Irinotecan** **(Nal-IRI)**	Ib/II	Nal-IRI 70 mg/m^2^ or 85 mg/m^2^ every 2 weeks	N/A	N/A	33.3%	Diarrhea (*n* = 5) Neutropenia (*n* = 4) Anemia (*n* = 2) Thrombocytopenia (*n* = 2)
**Belotecan**	II	Belotecan 0.5 mg/m^2^ 1–5q21	9.9	2.2	24%	Neutropenia (grade 3–4) (88%) Thrombocytopenia (40.0%)
	II	Belotecan 0.5 mg/m^2^ 1–5q21	6.5 sensitive 4.0 refractory	2.8 sensitive 1.5 refractory	20% sensitive 10% refractory	Neutropenia (54%) Thrombocytopenia (38%) Anemia (32%)
	IIb	Belotecan 0.5 mg/m^2^ 1–5q21 vs. Topotecan 1.5 mg/m^2^ 1–5q21	13.2 vs. 8.2 *p* = 0.018	4.8 vs. 3.8 *p* = 0.96	33 vs. 21% *p* = 0.09	Hematological disorders (≥10%) Neutropenia Thrombocytopenia Anaemia
**Amrubicin**	II	amrubicin (40 mg/m^2^ on days 1 through 3) or topotecan (1.0 mg/m^2^ on days 1 through 5) every 3 weeks	8.1 vs. 8.4	3.5 vs. 2.2	38% (95% CI, 20 to 56%) vs. 13% (95% CI, 1 to 25%)	Neutropenia (79%) Febrile Neutropenia (14%) Anemia (21%) Thrombocytopenia (28%)
	II	Amrubicin 40 mg/m^2^ on days 1 to 3 every 3 weeks	11.2	2.6 refractory 4.2 sensitive	52%	Neutropenia (83%) Thrombocytopenia (20%) Anemia (33%) Febrile neutropenia (5%)
	II	Amrubicin (40 mg/m^2^/d for 3 every 21 days) (NB: refractory patients)	6.0 (95% CI, 4.8 to 7.1)	3.2 (95% CI, 2.4 to 4.0)	21.3% (95% CI, 12.7 to 32.3%)	Neutropenia (67%) Thrombocytopenia (41%) Anemia (30%) Febrile neutropenia (12%)
	II	amrubicin (40 mg/m^2^ on days 1 through 3) or topotecan (1.0 mg/m^2^ on days 1 through 5) every 3 weeks NB: platinum sensitive	9.2 vs. 7.6	4.5 vs. 3.3	44 vs. 15%; *p* = 0.021	Neutropenia (61%) Thrombocytopenia (39%) Leukopenia (39%) Anemia (25%) Febrile neutropenia (10%)
	III	amrubicin (40 mg/m^2^ on days 1 through 3) or topotecan (1.0 mg/m^2^ on days 1 through 5) every 3 weeks	7.5 vs. 7.8 (HR = 0.880; *p* = 0.170)	4.1 vs. 3.5 (HR, 0.802; *p* = 0.018)	31.1 vs. 16.9% (odd ratio 2.223; *p* = 0.001)	Neutropenia (41%) Thrombocytopenia (21%) Anemia (16%) Infections (16%) Febrile neutropenia (10%) Cardiac disorders (5%) Need of transfusion (32%)
	III	cisplatin (60 mg/m^2^, day 1) amrubicin (40 mg/m^2^, days 1–3) vs. cisplatin and eto-poside (100 mg/m^2^, days 1–3) once every 21 days.	11.8 vs. 10.3 (*p* = 0.08)	6.8 vs. 5.7 months (*p* = 0.35)	69.8 vs. 57.3%	Neutropenia (54.4 %) Leukopenia (34.9 %) Thrombocytopenia (16.1 %)
**Temozolomide (TMZ)**	II	TMZ 75 mg/mq/die 1→21q28	NA	NA	22% sensitive 19% refractory	Thrombocytopenia and neutropenia (14%)
	II	TMZ 200 mg/mq/die 1→5 q28	1.8	5.8	12%	Anemia, thrombocytopenia and neutropenia (20%)
